# 2-(4-Fluoro­phen­yl)-3-methyl­sulfinyl-5-phenyl-1-benzofuran

**DOI:** 10.1107/S1600536810014613

**Published:** 2010-04-24

**Authors:** Hong Dae Choi, Pil Ja Seo, Byeng Wha Son, Uk Lee

**Affiliations:** a Department of Chemistry, Dongeui University, San 24 Kaya-dong Busanjin-gu, Busan 614-714, Republic of Korea; b Department of Chemistry, Pukyong National University, 599-1 Daeyeon 3-dong, Nam-gu, Busan 608-737, Republic of Korea

## Abstract

In the title mol­ecule, C_21_H_15_FO_2_S, the O atom and the methyl group of the methyl­sulfinyl substituent are situated on the opposite sides of the plane through the benzofuran fragment. The benzofuran ring plane makes dihedral angles of 28.63 (6) and 31.55 (5)° with the 4-fluoro­phenyl and phenyl rings, respectively. Weak C—H⋯F and C—H⋯O hydrogen bonds and inter­molecular C—H⋯π inter­actions are present in the crystal structure. The title crystal was refined as an inversion twin with a 0.39 (7):0.61 (7) domain ratio.

## Related literature

For the crystal structures of similar 3-alkyl­sulfanyl-2-(4-fluoro­phen­yl)-5-phenyl-1-benzofuran derivatives, see: Choi *et al.* (2009 ▶, 2010 ▶). For the pharmacological activity of benzofuran compounds, see: Aslam *et al.* (2006 ▶); Galal *et al.* (2009 ▶); Khan *et al.* (2005 ▶). For natural products with benzofuran rings, see: Akgul & Anil (2003 ▶); Soekamto *et al.* (2003 ▶). For hydrogen bonding, see: Desiraju & Steiner (1999 ▶).
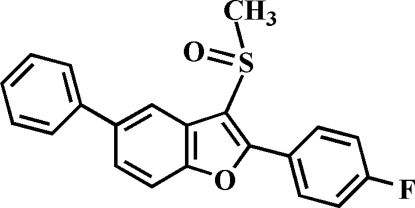


## Experimental

### 

#### Crystal data

C_21_H_15_FO_2_S
*M* *_r_* = 350.39
Monoclinic, 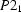

*a* = 10.148 (2) Å
*b* = 7.117 (1) Å
*c* = 11.991 (2) Å
β = 110.047 (2)°
*V* = 813.6 (2) Å^3^
*Z* = 2
Mo *K*α radiation
μ = 0.22 mm^−1^
*T* = 173 K
0.40 × 0.40 × 0.25 mm

#### Data collection

Bruker SMART APEXII CCD diffractometer
Absorption correction: multi-scan (*SADABS*; Bruker, 2009 ▶) *T*_min_ = 0.917, *T*_max_ = 0.947
4812 measured reflections
3226 independent reflections
3065 reflections with *I* > 2σ(*I*)
*R*_int_ = 0.019

#### Refinement

*R*[*F* ^2^ > 2σ(*F* ^2^)] = 0.032
*wR*(*F* ^2^) = 0.086
*S* = 1.06
3226 reflections
228 parameters
1 restraint
H-atom parameters constrained
Δρ_max_ = 0.31 e Å^−3^
Δρ_min_ = −0.21 e Å^−3^
Absolute structure: Flack (1983 ▶), 1308 Friedel pairs
Flack parameter: 0.39 (7)

### 

Data collection: *APEX2* (Bruker, 2009 ▶); cell refinement: *SAINT* (Bruker, 2009 ▶); data reduction: *SAINT*; program(s) used to solve structure: *SHELXS97* (Sheldrick, 2008 ▶); program(s) used to refine structure: *SHELXL97* (Sheldrick, 2008 ▶); molecular graphics: *ORTEP-3* (Farrugia, 1997 ▶) and *DIAMOND* (Brandenburg, 1998 ▶); software used to prepare material for publication: *SHELXL97*.

## Supplementary Material

Crystal structure: contains datablocks global, I. DOI: 10.1107/S1600536810014613/fb2185sup1.cif

Structure factors: contains datablocks I. DOI: 10.1107/S1600536810014613/fb2185Isup2.hkl

Additional supplementary materials:  crystallographic information; 3D view; checkCIF report

## Figures and Tables

**Table 1:** Hydrogen-bond geometry (Å, °) *Cg*1 and *Cg*2 are the centroids of the C15–C20 (5-phen­yl) and the C9–14 (4-fluoro­phen­yl) rings, respectively.

*D*—H⋯*A*	*D*—H	H⋯*A*	*D*⋯*A*	*D*—H⋯*A*
C18—H18⋯O2^i^	0.93	2.61	3.301 (3)	131
C21—H21*A*⋯O2^ii^	0.96	2.63	3.375 (3)	135
C21—H21*B*⋯F^iii^	0.96	2.55	3.478 (3)	164
C10—H10⋯*Cg*1^iv^	0.93	2.86	3.450 (2)	122
C13—H13⋯*Cg*2^iii^	0.93	2.81	3.417 (2)	124

Symmetry codes: (i) 
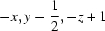
; (ii) 
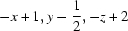
; (iii) 
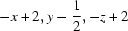
; (iv) 
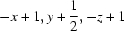
.

**Fig. 1.**
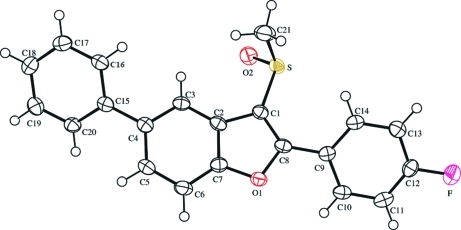
The title molecule with the atom numbering scheme. The displacement ellipsoids are drawn at the 50% probability level. The H atoms are depicted as small spheres of arbitrary radius.

**Fig. 2.**
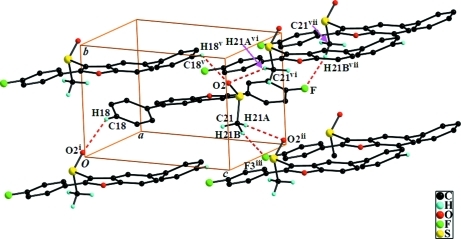
C—H···F and C—H···O interactions (dotted lines) in the crystal structure of the title compound. [Symmetry codes: (i) -x, y-1/2, -z+1; (ii) -x+1, y-1/2, -z+2; (iii) -x+2, y-1/2, -z+2; (v) -x, y+1/2, -z+1; (vi) -x+1, y+1/2, -z+2; (vii) -x+2, y+1/2, -z+2.]

**Fig. 3.**
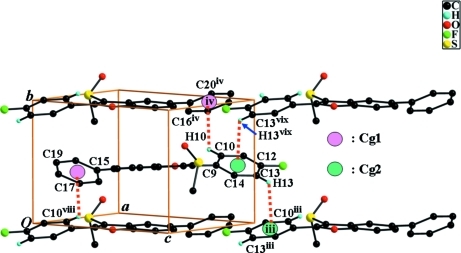
C—H···π-electron ring interactions (dotted lines) in the crystal structure of the title compound. Cg denotes the ring centroids. [Symmetry codes: (iii) -x+2, y-1/2, -z+2; (iv) -x+1, y+1/2, -z+1; (viii) -x+1, y-1/2, -z+1; (ix) -x+2, y+1/2, -z+2.]

## supplementary crystallographic information

### Comment

The compounds with the benzofuran skeleton show significant pharmacological activities such as fungicide (Aslam *et al.*, 2006), antitumor and antiviral (Galal *et al.*, 2009) and antimicrobial (Khan *et al.*, 2005) properties. These compounds are common in Nature (Akgul & Anil, 2003; Soekamto *et al.*, 2003). As a part of our ongoing studies of the effect of the side chain substituents on the solid state structures of 3-alkylsulfanyl-2-(4-fluorophenyl)-5-phenyl-1-benzofuran analogues (Choi *et al.*, 2009, 2010), we report the title crystal structure. The title molecule is depicted in Fig. 1.

The benzofuran ring is essentially planar, with a mean deviation of 0.006 (2) Å from the least-squares plane defined by the nine constituent atoms. In the molecule, the benzofuran plane makes dihedral angles of 28.63 (6) and 31.55 (5)° with the 4-fluorophenyl ring and the phenyl ring, respectively. The molecular packing (Fig. 2) is stabilized by an intermolecular C—H···F hydrogen bond between the methyl H atom and the fluorine (Tab. 1). There are also C—H···O interactions (Tab. 1 and Fig. 2) with geometrical parameters that are on the limit of their acceptance as true weak C—H···O hydrogen bonds (Desiraju & Steiner, 1999). The molecular packing (Fig. 3) is further stabilized by two intermolecular C—H···π-electron ring interactions: The first one between the 4-fluorophenyl H atom and the 5-phenyl ring, and the second one between the 4-fluorophenyl H atom and 4-fluorophenyl ring (Tab. 1).

### Experimental

77% 3-chloroperoxybenzoic acid (224 mg, 1.0 mmol) was added in small portions to a stirred solution of 2-(4-fluorophenyl)-3-methylsulfanyl-5-phenyl-1-benzofuran (301 mg, 0.9 mmol) in dichloromethane (30 ml) at 273 K. After having been stirred at room temperature for 4h, the mixture was washed with saturated sodium hydrogencarbonate solution and the organic layer was separated, dried over magnesium sulfate, filtered and concentrated at reduced pressure. The residue was purified by column chromatography (silica gel, hexane–ethyl acetate, 1:1 v/v) to afford the title compound as a colourless solid [yield 80%, m.p. 506–507 K; *R*_f_ = 0.59 (hexane–ethyl acetate, 1:1 v/v)]. Single crystals suitable for X-ray diffraction were prepared by slow evaporation of a solution of the title compound in chloroform at room temperature. The average crystal size was approximately 1.0 × 1.0 × 0.5 mm. (The measured crystal was cut from the larger one.) The crystals are colourless and soluble in polar solvents.

### Refinement

All the H atoms were positioned geometrically and refined using a riding model, with C—H = 0.93 Å for the aryl H atoms and 0.96 Å for the methyl H atoms, and with *U*_iso_(H) = 1.2*U*_eq_(C) for the aryl H atoms and 1.5*U*_eq_(C) for the methyl H atoms. 1308 Friedel pairs have been used in the refinement.

### Crystal data

**Table:**

C_21_H_15_FO_2_S	*F*(000) = 364
*M**_r_* = 350.39	*D*_x_ = 1.430 Mg m^−^^3^
Monoclinic, *P*2_1_	Melting point = 506–507 K
Hall symbol: P 2yb	Mo *K*α radiation, λ = 0.71073 Å
*a* = 10.148 (2) Å	Cell parameters from 3710 reflections
*b* = 7.117 (1) Å	θ = 2.3–28.3°
*c* = 11.991 (2) Å	µ = 0.22 mm^−^^1^
β = 110.047 (2)°	*T* = 173 K
*V* = 813.6 (2) Å^3^	Block, colourless
*Z* = 2	0.40 × 0.40 × 0.25 mm

### Data collection

**Table:**

Bruker SMART APEXII CCD diffractometer	3226 independent reflections
Radiation source: rotating anode	3065 reflections with *I* > 2σ(*I*)
graphite multilayer	*R*_int_ = 0.019
Detector resolution: 10.0 pixels mm^-1^	θ_max_ = 27.0°, θ_min_ = 1.8°
φ and ω scans	*h* = −8→12
Absorption correction: multi-scan (*SADABS*; Bruker, 2009)	*k* = −9→8
*T*_min_ = 0.917, *T*_max_ = 0.947	*l* = −15→14
4812 measured reflections	

### Refinement

**Table:**

Refinement on *F*^2^	Secondary atom site location: difference Fourier map
Least-squares matrix: full	Hydrogen site location: difference Fourier map
*R*[*F*^2^ > 2σ(*F*^2^)] = 0.032	H-atom parameters constrained
*wR*(*F*^2^) = 0.086	*w* = 1/[σ^2^(*F*_o_^2^) + (0.0516*P*)^2^ + 0.1094*P*] where *P* = (*F*_o_^2^ + 2*F*_c_^2^)/3
*S* = 1.06	(Δ/σ)_max_ < 0.001
3226 reflections	Δρ_max_ = 0.31 e Å^−^^3^
228 parameters	Δρ_min_ = −0.21 e Å^−^^3^
1 restraint	Absolute structure: Flack (1983), 1308 Friedel pairs
59 constraints	Flack parameter: 0.39 (7)
Primary atom site location: structure-invariant direct methods	

### Special details

**Table:**

Experimental. The measured sample has been cut from the larger crystal. The crystals, both the grown ones as well as the cut one, have not been examined under the polarization microscope.
Geometry. All esds (except the esd in the dihedral angle between two l.s. planes) are estimated using the full covariance matrix. The cell esds are taken into account individually in the estimation of esds in distances, angles and torsion angles; correlations between esds in cell parameters are only used when they are defined by crystal symmetry. An approximate (isotropic) treatment of cell esds is used for estimating esds involving l.s. planes.
Refinement. Refinement of F^2^ against ALL reflections. The weighted R-factor wR and goodness of fit S are based on F^2^, conventional R-factors R are based on F, with F set to zero for negative F^2^. The threshold expression of F^2^ > 2sigma(F^2^) is used only for calculating R-factors(gt) etc. and is not relevant to the choice of reflections for refinement. R-factors based on F^2^ are statistically about twice as large as those based on F, and R- factors based on ALL data will be even larger. The diffractions 1 0 0 and 0 0 1 as well as their equivalents have been excluded from the refinement because their respective intensities significantly differed from the calculated ones.

### Fractional atomic coordinates and isotropic or equivalent isotropic displacement parameters (Å^2^)

**Table:**

	*x*	*y*	*z*	*U*_iso_*/*U*_eq_	
S	0.55747 (5)	0.51643 (8)	0.86742 (4)	0.03009 (13)	
F	1.26845 (12)	0.4440 (2)	1.03910 (11)	0.0425 (3)	
O1	0.70363 (13)	0.4548 (2)	0.60162 (10)	0.0280 (3)	
O2	0.44274 (15)	0.6590 (2)	0.83904 (13)	0.0398 (4)	
C1	0.59124 (17)	0.4753 (3)	0.73449 (15)	0.0251 (4)	
C2	0.48587 (19)	0.4671 (3)	0.61728 (15)	0.0257 (4)	
C3	0.33954 (18)	0.4703 (3)	0.57251 (15)	0.0256 (4)	
H3	0.2883	0.4810	0.6234	0.031*	
C4	0.27128 (18)	0.4572 (3)	0.44981 (15)	0.0255 (4)	
C5	0.35234 (19)	0.4441 (3)	0.37470 (16)	0.0286 (4)	
H5	0.3061	0.4352	0.2932	0.034*	
C6	0.4975 (2)	0.4439 (3)	0.41758 (16)	0.0317 (4)	
H6	0.5495	0.4366	0.3672	0.038*	
C7	0.56102 (19)	0.4553 (3)	0.53979 (16)	0.0264 (4)	
C8	0.71883 (19)	0.4670 (3)	0.72036 (15)	0.0255 (4)	
C9	0.86341 (18)	0.4633 (3)	0.80353 (15)	0.0242 (4)	
C10	0.97478 (18)	0.5278 (3)	0.76948 (16)	0.0278 (4)	
H10	0.9566	0.5745	0.6932	0.033*	
C11	1.11179 (18)	0.5224 (3)	0.84845 (17)	0.0317 (4)	
H11	1.1857	0.5663	0.8265	0.038*	
C12	1.13528 (19)	0.4503 (3)	0.96030 (17)	0.0291 (4)	
C13	1.0289 (2)	0.3813 (3)	0.99652 (17)	0.0288 (4)	
H13	1.0484	0.3314	1.0722	0.035*	
C14	0.89299 (19)	0.3886 (3)	0.91741 (16)	0.0268 (4)	
H14	0.8201	0.3432	0.9402	0.032*	
C15	0.11547 (19)	0.4540 (3)	0.39950 (15)	0.0250 (4)	
C16	0.0356 (2)	0.3732 (3)	0.46221 (16)	0.0281 (4)	
H16	0.0806	0.3231	0.5373	0.034*	
C17	−0.1097 (2)	0.3669 (3)	0.41369 (17)	0.0313 (4)	
H17	−0.1610	0.3139	0.4568	0.038*	
C18	−0.1788 (2)	0.4392 (3)	0.30148 (18)	0.0323 (4)	
H18	−0.2760	0.4326	0.2686	0.039*	
C19	−0.10197 (19)	0.5216 (3)	0.23840 (16)	0.0315 (4)	
H19	−0.1478	0.5718	0.1635	0.038*	
C20	0.04378 (18)	0.5288 (3)	0.28756 (15)	0.0277 (4)	
H20	0.0944	0.5848	0.2448	0.033*	
C21	0.4775 (2)	0.2943 (4)	0.8756 (2)	0.0421 (6)	
H21A	0.4490	0.2925	0.9441	0.063*	
H21B	0.5438	0.1952	0.8817	0.063*	
H21C	0.3970	0.2765	0.8053	0.063*	

### Atomic displacement parameters (Å^2^)

**Table:**

	*U*^11^	*U*^22^	*U*^33^	*U*^12^	*U*^13^	*U*^23^
S	0.0302 (2)	0.0398 (3)	0.02136 (19)	0.0062 (2)	0.01031 (15)	−0.0019 (2)
F	0.0287 (6)	0.0505 (8)	0.0447 (7)	−0.0009 (6)	0.0080 (5)	0.0011 (6)
O1	0.0297 (7)	0.0328 (7)	0.0243 (6)	0.0016 (6)	0.0128 (5)	0.0010 (6)
O2	0.0390 (8)	0.0470 (10)	0.0350 (8)	0.0117 (7)	0.0148 (7)	−0.0033 (7)
C1	0.0285 (9)	0.0257 (11)	0.0232 (8)	0.0025 (7)	0.0116 (7)	−0.0012 (7)
C2	0.0336 (9)	0.0226 (10)	0.0228 (8)	0.0019 (7)	0.0120 (7)	0.0007 (7)
C3	0.0306 (9)	0.0244 (11)	0.0253 (8)	0.0003 (7)	0.0142 (7)	−0.0004 (7)
C4	0.0306 (9)	0.0213 (9)	0.0250 (8)	−0.0008 (8)	0.0100 (7)	0.0015 (8)
C5	0.0361 (10)	0.0289 (10)	0.0224 (8)	−0.0012 (8)	0.0122 (7)	−0.0009 (8)
C6	0.0364 (10)	0.0369 (11)	0.0269 (9)	0.0023 (9)	0.0173 (8)	0.0011 (9)
C7	0.0292 (9)	0.0247 (9)	0.0285 (9)	0.0011 (8)	0.0140 (7)	0.0003 (8)
C8	0.0322 (9)	0.0220 (10)	0.0253 (8)	0.0005 (7)	0.0135 (7)	0.0017 (7)
C9	0.0264 (9)	0.0198 (9)	0.0289 (8)	0.0020 (7)	0.0129 (7)	−0.0005 (8)
C10	0.0346 (9)	0.0241 (9)	0.0304 (8)	0.0022 (9)	0.0184 (7)	0.0027 (9)
C11	0.0311 (9)	0.0265 (10)	0.0439 (10)	−0.0006 (9)	0.0211 (8)	−0.0002 (10)
C12	0.0249 (9)	0.0264 (10)	0.0354 (9)	0.0025 (8)	0.0094 (7)	−0.0021 (9)
C13	0.0337 (11)	0.0249 (10)	0.0289 (9)	0.0027 (8)	0.0121 (8)	0.0015 (8)
C14	0.0292 (10)	0.0242 (10)	0.0310 (9)	0.0007 (7)	0.0154 (8)	0.0016 (8)
C15	0.0330 (9)	0.0196 (9)	0.0237 (8)	−0.0035 (8)	0.0114 (7)	−0.0042 (8)
C16	0.0374 (11)	0.0254 (10)	0.0234 (8)	−0.0021 (8)	0.0126 (8)	−0.0002 (8)
C17	0.0352 (10)	0.0285 (11)	0.0351 (10)	−0.0046 (8)	0.0183 (8)	−0.0027 (9)
C18	0.0265 (9)	0.0319 (11)	0.0385 (10)	−0.0017 (8)	0.0108 (8)	−0.0040 (9)
C19	0.0358 (9)	0.0270 (10)	0.0288 (8)	0.0016 (9)	0.0073 (7)	0.0011 (9)
C20	0.0342 (9)	0.0234 (9)	0.0273 (8)	−0.0040 (9)	0.0130 (7)	0.0017 (9)
C21	0.0451 (13)	0.0481 (15)	0.0405 (12)	0.0055 (11)	0.0242 (10)	0.0131 (11)

### Geometric parameters (Å, °)

**Table:**

S—O2	1.4931 (16)	C10—H10	0.9300
S—C1	1.7660 (17)	C11—C12	1.379 (3)
S—C21	1.795 (2)	C11—H11	0.9300
F—C12	1.359 (2)	C12—C13	1.385 (3)
O1—C8	1.381 (2)	C13—C14	1.381 (3)
O1—C7	1.382 (2)	C13—H13	0.9300
C1—C8	1.364 (2)	C14—H14	0.9300
C1—C2	1.446 (2)	C15—C20	1.395 (3)
C2—C7	1.392 (2)	C15—C16	1.404 (3)
C2—C3	1.395 (2)	C16—C17	1.388 (3)
C3—C4	1.398 (2)	C16—H16	0.9300
C3—H3	0.9300	C17—C18	1.386 (3)
C4—C5	1.416 (2)	C17—H17	0.9300
C4—C15	1.487 (2)	C18—C19	1.388 (3)
C5—C6	1.383 (3)	C18—H18	0.9300
C5—H5	0.9300	C19—C20	1.393 (2)
C6—C7	1.386 (3)	C19—H19	0.9300
C6—H6	0.9300	C20—H20	0.9300
C8—C9	1.464 (2)	C21—H21A	0.9600
C9—C14	1.399 (2)	C21—H21B	0.9600
C9—C10	1.404 (2)	C21—H21C	0.9600
C10—C11	1.389 (2)		
			
O2—S—C1	106.52 (9)	C12—C11—H11	120.9
O2—S—C21	106.22 (10)	C10—C11—H11	120.9
C1—S—C21	97.72 (10)	F—C12—C11	119.20 (16)
C8—O1—C7	106.22 (13)	F—C12—C13	117.85 (18)
C8—C1—C2	107.14 (15)	C11—C12—C13	122.95 (18)
C8—C1—S	127.27 (14)	C14—C13—C12	118.27 (18)
C2—C1—S	125.19 (13)	C14—C13—H13	120.9
C7—C2—C3	119.90 (16)	C12—C13—H13	120.9
C7—C2—C1	105.03 (15)	C13—C14—C9	120.97 (17)
C3—C2—C1	135.07 (16)	C13—C14—H14	119.5
C2—C3—C4	118.80 (15)	C9—C14—H14	119.5
C2—C3—H3	120.6	C20—C15—C16	117.73 (17)
C4—C3—H3	120.6	C20—C15—C4	121.06 (15)
C3—C4—C5	119.18 (16)	C16—C15—C4	121.20 (16)
C3—C4—C15	120.16 (15)	C17—C16—C15	120.94 (18)
C5—C4—C15	120.66 (16)	C17—C16—H16	119.5
C6—C5—C4	122.70 (17)	C15—C16—H16	119.5
C6—C5—H5	118.7	C18—C17—C16	120.43 (18)
C4—C5—H5	118.7	C18—C17—H17	119.8
C5—C6—C7	116.31 (16)	C16—C17—H17	119.8
C5—C6—H6	121.8	C17—C18—C19	119.61 (18)
C7—C6—H6	121.8	C17—C18—H18	120.2
O1—C7—C6	126.11 (16)	C19—C18—H18	120.2
O1—C7—C2	110.78 (15)	C18—C19—C20	119.88 (17)
C6—C7—C2	123.10 (17)	C18—C19—H19	120.1
C1—C8—O1	110.83 (15)	C20—C19—H19	120.1
C1—C8—C9	133.51 (16)	C19—C20—C15	121.40 (17)
O1—C8—C9	115.64 (14)	C19—C20—H20	119.3
C14—C9—C10	118.91 (16)	C15—C20—H20	119.3
C14—C9—C8	120.10 (15)	S—C21—H21A	109.5
C10—C9—C8	120.96 (16)	S—C21—H21B	109.5
C11—C10—C9	120.69 (17)	H21A—C21—H21B	109.5
C11—C10—H10	119.7	S—C21—H21C	109.5
C9—C10—H10	119.7	H21A—C21—H21C	109.5
C12—C11—C10	118.19 (16)	H21B—C21—H21C	109.5
			
O2—S—C1—C8	133.81 (19)	C7—O1—C8—C9	178.29 (16)
C21—S—C1—C8	−116.67 (19)	C1—C8—C9—C14	27.8 (3)
O2—S—C1—C2	−38.02 (19)	O1—C8—C9—C14	−150.11 (18)
C21—S—C1—C2	71.50 (18)	C1—C8—C9—C10	−154.5 (2)
C8—C1—C2—C7	−0.3 (2)	O1—C8—C9—C10	27.6 (3)
S—C1—C2—C7	172.91 (16)	C14—C9—C10—C11	−1.7 (3)
C8—C1—C2—C3	−179.7 (2)	C8—C9—C10—C11	−179.48 (19)
S—C1—C2—C3	−6.5 (3)	C9—C10—C11—C12	0.7 (3)
C7—C2—C3—C4	1.4 (3)	C10—C11—C12—F	−179.86 (19)
C1—C2—C3—C4	−179.3 (2)	C10—C11—C12—C13	0.8 (3)
C2—C3—C4—C5	−1.0 (3)	F—C12—C13—C14	179.42 (18)
C2—C3—C4—C15	178.04 (17)	C11—C12—C13—C14	−1.2 (3)
C3—C4—C5—C6	−0.1 (3)	C12—C13—C14—C9	0.1 (3)
C15—C4—C5—C6	−179.11 (19)	C10—C9—C14—C13	1.3 (3)
C4—C5—C6—C7	0.7 (3)	C8—C9—C14—C13	179.05 (18)
C8—O1—C7—C6	−179.6 (2)	C3—C4—C15—C20	149.0 (2)
C8—O1—C7—C2	−0.1 (2)	C5—C4—C15—C20	−32.0 (3)
C5—C6—C7—O1	179.1 (2)	C3—C4—C15—C16	−31.9 (3)
C5—C6—C7—C2	−0.3 (3)	C5—C4—C15—C16	147.1 (2)
C3—C2—C7—O1	179.75 (16)	C20—C15—C16—C17	0.5 (3)
C1—C2—C7—O1	0.3 (2)	C4—C15—C16—C17	−178.57 (19)
C3—C2—C7—C6	−0.8 (3)	C15—C16—C17—C18	0.6 (3)
C1—C2—C7—C6	179.71 (19)	C16—C17—C18—C19	−1.3 (3)
C2—C1—C8—O1	0.2 (2)	C17—C18—C19—C20	0.8 (3)
S—C1—C8—O1	−172.79 (14)	C18—C19—C20—C15	0.3 (3)
C2—C1—C8—C9	−177.7 (2)	C16—C15—C20—C19	−1.0 (3)
S—C1—C8—C9	9.2 (3)	C4—C15—C20—C19	178.14 (19)
C7—O1—C8—C1	−0.1 (2)		

### Hydrogen-bond geometry (Å, °)

**Table:**

Cg1 and Cg2 are the centroids of the C15–C20 (5-phenyl) and the C9–14 (4-fluorophenyl) rings, respectively.

**Table:**

*D*—H···*A*	*D*—H	H···*A*	*D*···*A*	*D*—H···*A*
C18—H18···O2^i^	0.93	2.61	3.301 (3)	131
C21—H21A···O2^ii^	0.96	2.63	3.375 (3)	135
C21—H21B···F^iii^	0.96	2.55	3.478 (3)	164
C10—H10···Cg1^iv^	0.93	2.86	3.450 (2)	122
C13—H13···Cg2^iii^	0.93	2.81	3.417 (2)	124

Symmetry codes: (i) −*x*, *y*−1/2, −*z*+1; (ii) −*x*+1, *y*−1/2, −*z*+2; (iii) −*x*+2, *y*−1/2, −*z*+2; (iv) −*x*+1, *y*+1/2, −*z*+1.
